# Physician Knowledge, Attitudes, and Practices Regarding Viral Exanthem Diagnosis and Mandatory Reporting Requirements in Major Metropolitan Regions of Florida, USA

**DOI:** 10.7759/cureus.117145

**Published:** 2026-09-29

**Authors:** Winnie Chen, Sarah Ballarin, Maria Fioletova, Chanakya R Bhosale, Thomas Matthews, Cyril Blavo, Jayson Forbes, Anna K Potter

**Affiliations:** 1 Foundational Science, Nova Southeastern University Dr. Kiran C. Patel College of Osteopathic Medicine, Fort Lauderdale, USA; 2 Foundational Science, Nova Southeastern University Dr. Kiran C. Patel College of Osteopathic Medicine, Clearwater, USA; 3 Public Health, Nova Southeastern University Dr. Kiran C. Patel College of Osteopathic Medicine, Fort Lauderdale, USA

**Keywords:** knowledge attitudes and practices (kap), mandatory reporting, measles, physician knowledge, roseola, rubella, vaccine-preventable diseases, viral exanthems

## Abstract

Background

Viral exanthems (VEs), including vaccine-preventable infections such as measles and rubella and non-vaccine-preventable infections such as roseola, remain clinically relevant. Declining vaccination rates and recent outbreaks have increased the importance of timely diagnosis and mandatory reporting. In addition, the relative rarity of vaccine-preventable diseases in modern clinical practice may reduce physician familiarity with these infections.

Methods

This IRB-exempt cross-sectional study assessed the knowledge, attitudes, and practices (KAP) of Florida physicians regarding VE diagnosis and reporting requirements using a 19-question Research Electronic Data Capture (REDCap; Vanderbilt University, Nashville, USA) survey. Participants included physicians and residents in family medicine, pediatrics, internal medicine, and infectious disease specialties. Statistical analyses included analysis of variance (ANOVA), multivariate ANOVA (MANOVA), Tukey post hoc testing, and linear regression.

Results

A total of 162 physicians completed the survey, with 146 complete responses included in inferential analyses. Pediatricians demonstrated the highest overall knowledge scores (82.66%), followed by internal medicine (76.39%) and family medicine physicians (73.26%). Overall knowledge differences among specialties were significant (F(2,143) = 3.31, p = 0.039), with pediatricians scoring significantly higher than family medicine physicians. No significant specialty differences were identified when vignette-based diagnostic or mandatory reporting questions were analyzed independently. Most physicians, 148 (91.36%), expressed interest in additional training on VE diagnosis and management. Greater physician confidence was significantly associated with higher diagnostic knowledge (β = 10.73, p < 0.001) and reporting knowledge (β = 9.68, p < 0.001). Roseola was the most commonly diagnosed VE in clinical practice, followed by measles and rubella.

Conclusion

Florida physicians demonstrated moderate-to-high knowledge of VE diagnosis and reporting requirements; however, notable gaps remained, particularly in differentiating rubella from similar exanthems. Interpretation is limited by the non-random respondent sample, uneven speciality representation, and potential nonresponse bias. Pediatricians outperformed family medicine physicians, although deficiencies were observed across specialties. These findings identify areas in which additional physician education may be beneficial, particularly regarding the clinical differentiation of viral exanthems and mandatory reporting requirements. As vaccination rates decline and outbreaks increase, improving early recognition and reporting of VE remains important for outbreak prevention and public health preparedness.

## Introduction

Viral exanthems (VEs) are rashes associated with systemic viral infections such as measles, rubella, and roseola [1]. They are often characterized by a diffuse eruption of macules and papules that can be erythematous in nature [2]. VEs can be differentiated based on distinct patterns; however, definitive diagnosis cannot always be made solely on the rash’s appearance and symptoms. It is important for clinicians to incorporate a comprehensive patient history, including travel from endemic areas, vaccination records, prodromal symptoms, and potential exposure history, to aid diagnostic accuracy. While these infections are typically self-limiting, atypical or severe patterns can present in immunocompromised patients, children, and pregnant women [3]. Despite the availability of safe and tested vaccine options, VE, such as measles, was the cause of 142,300 deaths worldwide in 2018, with the majority of cases found among young children and immunocompromised adults, driven by increased travel from endemic areas, limited vaccine availability in under-resourced areas, and declining vaccination rates in regions where they were previously eliminated, indicating the continuing public health importance of these viruses [4, 5].

The measles virus is a paramyxovirus belonging to the genus *Morbillivirus* [6]. The measles virus is a highly contagious viral infection transmitted via respiratory or aerosolized droplets. It uses host receptors including CD150 (SLAMF1) to infect susceptible immune cells of the respiratory tract, which then transport the virus to the lymphoid cells, further causing systemic disease and contributing to its high virulence and contagiousness [7]. After the virus gains entry, there is an incubation period of up to 3 weeks, which is followed by the prodromal phase of a high fever, often greater than 39 degrees Celsius [6]. Other early symptoms are cough, malaise, and the pathognomonic Koplik spots, which are small, white spots located on the buccal mucosa. The rash in measles begins on the face before spreading to the rest of the body. Due to overlapping symptoms with the common cold and other illnesses that have symptoms of a fever and rash, measles can be misdiagnosed [8]. Currently, the preferred method of diagnosing measles is nucleic acid amplification test (NAAT). Serologic IgM can be used to make the diagnosis, but clinical caution should be exercised as patients within 72 hours of developing a rash can return false-negative serology up to 20% of the time [9]. Rubella virus is a non-vector-borne member of the family *Togaviridae* and the sole member of the genus *Rubivirus *[10]. Rubella virus contains RNA, which is surrounded by a capsid and a lipoprotein envelope. Rubella is asymptomatic in 25% to 50% of young children with an incubation period of 2 to 3 weeks. Early symptoms are a mild fever, malaise, lymphadenopathy, and sore throat that precede a pinpoint erythematous maculopapular rash. The classic rubella rash starts on the face and neck and proceeds to spread to the trunk and lower extremities over the course of 3 days [11]. Complications such as congenital rubella syndrome (CRS), which affects newborns when a pregnant woman is exposed to the rubella virus, manifest in the triad of cataracts, congenital defects, and deafness. Since clinical manifestations can range from mild to atypical presentations, clinical diagnosis is made not only on the symptoms, but also serological testing for rubella-specific IgM and/or IgG antibodies remains the gold standard of diagnosis [9].

Roseola infantum, also known as exanthema subitum or sixth disease, is predominantly caused by infection with human herpesvirus 6 (HHV-6), or, less commonly, by human herpesvirus 7 (HHV-7) [12]. About ninety percent of cases occur in children younger than two years. Its clinical course begins with a high fever of up to 40 degrees Celsius, lasting 3 to 5 days, followed classically by a maculopapular erythematous rash originating on the trunk as the fever resolves. A complication of roseola infantum is acute febrile seizures due to its ability to cross the blood-brain barrier. Roseola infantum is a clinical diagnosis without any need for serology. Unlike measles and rubella, roseola has no vaccine available, but it continues to affect young children predominantly, accounting for 10 to 45% of febrile illnesses in infants, warranting the need for proper public health measures to avoid spreading the virus [12].

Although cases of VE, primarily measles, mumps, and rubella, have drastically decreased in the United States due to high vaccination rates, outbreaks and cases continue to occur in areas with high travel from endemic or under-resourced areas, including the State of Florida. These cases pose a public health threat in regions where proper public health measures, including vaccine coverage, continue to fall. While vaccine-preventable VE rates have plummeted following implementation of mandatory school vaccines in 1997 for middle school entry, Florida was the first state in the United States to move to end all mandates for vaccine requirements for children and adults on September 3rd, 2025, with planned removal of select vaccine mandates in January 2026 [13, 14, 15]. As of August 5th, 2025, there have been 1356 confirmed measles cases in 41 jurisdictions, with 32 outbreaks reported in 2025 compared to 16 outbreaks in 2024 [16]. Of the 1356 cases in 2025, 92% were unvaccinated or had unknown vaccine history, the majority under age 20 years old, with most of the cases being in Texas (800+) [16]. In 2024-2025, Florida had 30 confirmed cases of measles [17]. People are most protected through herd immunity when over 95% of the community is vaccinated; however, Measles, Mumps, and Rubella (MMR) vaccination coverage among kindergarteners in the USA has decreased to 92.7% in 2023-2024 from 95.2% during 2019-2020 [16]. In the United States, vaccines are readily available, but drivers of vaccine hesitancy include misinformation about side effects, reduced vaccination rates during and after the COVID-19 pandemic, health disparities among underserved groups in rural and urban settings, and a lack of awareness regarding the severity of infection, leading to potential public health risks [18]. The current and recent outbreaks of VE stress the importance of proper public health strategies to prevent future outbreaks, including continuing education of healthcare professionals, especially in primary care, pediatrics, and family medicine, where patients are more likely to present with complications of VE.

Previous studies have examined healthcare professionals’ knowledge of vaccine-preventable diseases and measles. However, few studies have looked at both viral exanthem diagnosis and mandatory reporting requirements. This gap helped support the need for the present study. Thus, in light of current circumstances, it is increasingly critical for physicians and healthcare workers to remain vigilant in recognizing vaccine-preventable infectious diseases to aid in public health measures and avoid further outbreaks of these diseases. The purpose of this study is to determine the current knowledge, attitudes, and practices among practicing physicians regarding viral exanthem diagnosis and their mandatory reporting requirements in various metropolitan areas of Florida, USA. The primary objective was to assess Florida physicians’ knowledge of the clinical differentiation and mandatory reporting requirements of measles, rubella, and roseola. Secondary objectives were to compare knowledge across medical specialties and examine associations between self-reported confidence and knowledge scores. We hypothesize that our data will reveal differences between physicians of different specialties or of different levels of exposure to these viruses.

## Materials and methods

Overall study design

This IRB-exempt cross-sectional study utilized an online 19-question survey that consisted of six demographic/professional questions, six knowledge questions, five attitude questions, and two practice questions. The methodology for this study was closely modeled after that of a preliminary study [19], with modifications to address the specific aims of the present investigation. The modified survey focused on viral exanthem diagnosis, mandatory reporting requirements, physician confidence, and self-reported clinical practices. The modified instrument did not undergo formal content validation or reliability testing. See Appendices for the list of questions utilized. The inclusion criteria for this study were actively licensed physicians (DO or MD) or residents with a medical training license. Specialties of focus included primary care, family medicine/practice, pediatrics, internal medicine, and infectious disease. Overall, the questions tested participants' knowledge of viral exanthem diagnosis, treatment, and risk factors. No intervention or treatment was used in this study.

Participants and specialties

Prior research has established deficiencies in knowledge of VE amongst physicians. However, no studies specifically measured responses from physicians practicing in the state of Florida, and no studies have examined mandatory reporting requirements in the United States. Potential participants were first identified utilizing the Florida Department of Health public practitioner profile database of healthcare professionals, filtered by license in “active” status as of November 2024, county, and specialty. A broad county-based recruitment strategy was used to identify eligible physicians. The specialties of choice were family medicine, internal medicine, pediatrics, and infectious disease. Emails of 1085 family medicine, 1265 pediatric, and 491 infectious disease doctors were collected. Internal medicine physicians were not independently targeted during the initial email recruitment process. Responders classified as internal medicine were identified based on reported specialty or subspecialty information on the survey. Survey responses were collected from January 2025 to July 2025. Participants were recruited from the Florida Department of Health practitioner database using predefined specialty and county criteria. This was not a probability-based random sample of all eligible Florida physicians. Counties were broadly grouped into three geographic regions: North, Central, or South Florida. South Florida comprised Miami-Dade, Monroe, Broward, Collier, Palm Beach, Hendry, Lee, Martin, Glades, and Charlotte counties. Central Florida comprised St. Lucie, Okeechobee, Highlands, Desoto, Hardee, Sarasota, Manatee, Indian River, Brevard, Osceola, Polk, Hillsborough, Pinellas, Orange, Seminole, Lake, Sumter, Pasco, Hernando, Citrus, and Volusia counties. North Florida comprised Flagler, Marion, Levy, Putnam, Alachua, Gilchrist, Dixie, St. Johns, Clay, Bradford, Union, Columbia, Suwannee, Lafayette, Taylor, Duval, Nassau, Baker, Hamilton, Madison, Jefferson, Leon, Wakulla, Gadsden, Liberty, Franklin, Gulf, Calhoun, Jackson, Holmes, Washington, Bay, Walton, Okaloosa, Santa Rosa, and Escambia counties.

Survey instrument

In order to ensure the anonymity and safety of survey responses, the secure REDCap (Vanderbilt University, Nashville, USA) survey platform was utilized to distribute our survey and capture data. REDCap facilitated sending survey invites, retrieving responses, and initiating data analyses. Surveys were sent out randomly, on a weekly basis, and this was repeated up to ten times if no response was received. The survey consisted of six vignette-style knowledge questions: three questions that addressed diagnosing a viral exanthem, and the other three questions focused on mandatory reporting of VE. There were five attitude questions: regarding the clinical process of diagnosis, physician training on diagnosing VE, misdiagnosis/underdiagnosis of VE, confidence in diagnosing VE, and confidence of properly reporting VE. Two practice questions assessed: perceived childhood vaccination rates among respective patient populations, and what VE physicians have diagnosed.

Data analysis

For each question type, a sample proportion (%) was calculated by dividing the number of positive responses by the total number of survey responses. Overall knowledge scores were calculated as the percentage of correctly answered items across all six vignette-style knowledge questions. Each question was scored as correct (1) or incorrect (0). Vignette knowledge scores were calculated from the three diagnostic vignette questions only; mandatory reporting knowledge scores were calculated from the three mandatory reporting questions only. Scores are expressed as percentages.

Specific statistical analysis

Survey responses regarding practicing specialty were consolidated into either family medicine, internal medicine, or pediatrics. Infectious diseases and other subspecialties of internal medicine were sorted under internal medicine unless otherwise specified by the surveyor. Analysis of variance (ANOVA) was conducted in the comparison of knowledge and attitude questions by specialty. This was followed by pairwise comparisons to determine significance between groups. A multivariate analysis of variance (MANOVA) was used to compare vignette-style diagnosis and mandatory reporting knowledge questions. The relationships between vignette-style diagnosis knowledge questions and confidence attitude questions were analyzed through linear regression.

Sample size and statistical power

The sample size was not established through an a priori power calculation but was determined by the recruitment framework, where physicians were identified from the Florida Department of Health database and contacted by specialty and county, yielding 162 respondents used for descriptive reporting and 146 complete responses used for statistical analyses. A complete response was defined as a survey response containing all variables required for the primary inferential analyses, including specialty classification and responses to all six knowledge questions. A sensitivity analysis was conducted to compare overall knowledge scores by specialty. At an alpha of 0.05 and a target power of 0.80, the analytic sample of 146 was used to estimate the minimum detectable effect size. Separately, post hoc power based on the observed between-specialty effect was approximately 0.63. The observed difference was significant (F(2,143) = 3.31, p = 0.039). These values reflect different aspects of statistical power: the target power was used to characterize the detectable effect size for the available sample, whereas the post hoc power reflects the observed effect. The study may therefore have been underpowered to detect smaller between-group differences, and nonsignificant subgroup comparisons should be interpreted cautiously.

IRB and human subjects disclaimer

The research was reviewed and was exempted by the Institutional Review Board of the Dr. Kiran C. Patel College of Osteopathic Medicine at Nova Southeastern University (NSU-KPCOM) on 12/20/2024, IRB number: 2024-655. The questionnaire included a prompt to receive informed consent electronically.

## Results

Demographics

A total of 162 practicing physicians across Florida completed the survey. Among these, 146 participants provided complete data, representing various specialties such as family medicine (FM) 48 (29.6%), internal medicine (IM) 24 (14.8%), and pediatrics 74 (45.7%). Related subspecialties 16 (9.9%) were included in the overall descriptive demographic reporting, but were excluded from inferential analysis of knowledge and attitude-based questions. Of 162 responses, reported durations of practice among participating physicians spanned from less than 1 year to 53 years. The mean number of years in practice was 21.96 years with a standard deviation (SD) of 13.72. The median and mode number of years in practice was 20 years. Represented training backgrounds of participants included MD 118 (72.8%), DO 40 (24.7%), and physician residents 4 (2.47%) (Table 1). With the exception of specialty, demographic data were excluded from statistical analysis due to incomplete survey responses. Additionally, these elements were deemed to be outside the scope of the primary research question.

**Table 1:** Demographics table for the physician responses Demographic and professional characteristics of physician respondents (162), including years in practice, professional credential or training status, Florida region, medical specialty, geographic practice location, and practice setting.

Characteristics	Category	# of Physicians	% of Physicians
Years Practising			
	5 and under	29	18%
	5–10	13	8%
	11–20	41	25%
	21–30	39	24%
	31–40	17	10%
	40 and over	23	14%
License			
	MD	118	73%
	DO	40	25%
	Resident	4	2%
Region			
	South Florida	66	41%
	Central Florida	44	27%
	North Florida	36	22%
	Not reported	16	10%
Specialty			
	Pediatrics	74	46%
	Family Medicine (FM)	48	30%
	Internal Medicine (IM)	24	15%
	Not reported	16	10%
Location			
	Urban	67	41%
	Suburban	64	40%
	Rural	12	7%
	Urban, Suburban	13	8%
	Suburban, Rural	3	2%
	Urban, Suburban, Rural	3	2%
Practice Setting			
	Academic	53	29%
	Community Hospital/Clinic	51	28%
	Private Practice	60	32%
	Telemedicine	9	5%
	Other	12	6%

A total of 19 counties were represented based on participants' self-reported practice locations. The counties, listed from most to least represented, were: Miami-Dade 30 (21.4%), Duval 19 (13.6%), Broward 18 (12.9%), Hillsborough 18 (12.9%), Orange 17 (12.1%), Palm Beach 9 (6.4%), Escambia 5 (3.6%), Leon 4 (2.9%), St. Johns 3 (2.1%), Pinellas 3 (2.1%), Lee 3 (2.1%), Volusia 2 (1.4%), Polk 2 (1.4%), Marion 2 (1.4%), St. Lucie 1 (0.7%), Seminole 1 (0.7%), Pasco 1 (0.7%), Collier 1 (0.7%), and Clay 1 (0.7%) (Figure 1).

**Figure 1:**
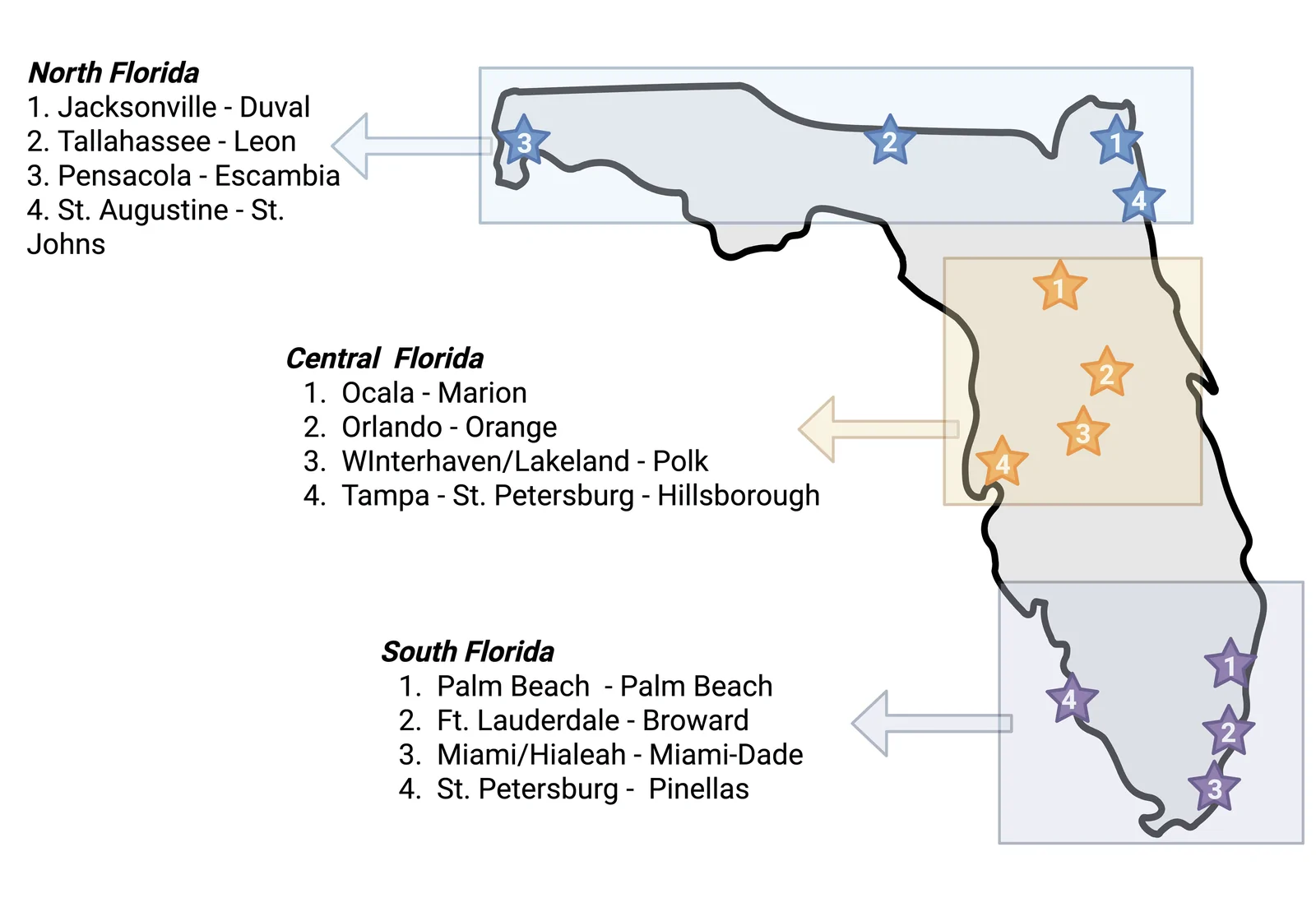
Map of Florida with major urban areas targeted in the study. Map of Florida representing three major areas of interest, which include North, South, and Central Florida. Within each area, at least four major counties were identified in which results were grouped and compared. Map created in BioRender.com (BioRender, Toronto, Canada) with a license to Chanakya R. Bhosale, available at https://app.biorender.com/citation/6a3c6d8f82c770f6661da4a7, licensed under CC BY 4.0.

Of 162 respondents, geographic practice settings reported by participants included urban 83 (51.2%), suburban 83 (51.2%), and rural 18 (11.1%). Business practice settings included academic 53 (28.6%), community hospital or clinic 51 (27.6%), private practice 60 (32.4%), telemedicine 9 (4.9%), and other 12 (6.49%). Participants were permitted to select multiple categories for both setting questions to most accurately reflect physicians’ practice environments (Table 1).

Overall knowledge performance

Across 146 full-response participants, overall knowledge scores for vignette and mandatory reporting questions ranged from 16.67% to 100% with a mean of 78.54% (SD = 20.53). Specialty-specific knowledge means (M) showed that pediatricians 74 (50.7%) scored the highest (M = 82.66%, SD = 16.42), followed by internal medicine physicians 24 (16.4%) (M = 76.39%, SD = 25.02), and family medicine physicians 48 (32.9%) (M = 73.26%, SD = 22.74) (Table 2). A one-way analysis of variance (ANOVA) demonstrated that differences in overall knowledge scores between specialties were significant (F(2,143) = 3.31, p = 0.04) (Table 3). Furthermore, the Tukey post hoc comparisons revealed that only the pediatricians group scored significantly higher than family medicine physicians by 9.39% (p = 0.04) (Table 3). There were no significant differences between pediatrics and internal medicine, or between family medicine and internal medicine.

**Table 2:** Overall Knowledge Scores by Specialty Distribution of overall viral exanthem knowledge scores among physician respondents, stratified by medical specialty (146), including the number of respondents, mean percentage score, standard deviation, and observed minimum and maximum scores.

Specialty	n	Mean (%)	SD (%)	Min (%)	Max (%)
Family Medicine	48	73.26	22.74	16.67	100
Internal Medicine	24	76.39	25.02	16.67	100
Pediatrics	74	82.66	16.42	33.33	100
Total	146	78.54	20.53	16.67	100

**Table 3:** ANOVA Results One-way ANOVA and Tukey post-hoc comparisons of physician knowledge and confidence scores across specialties. Overall knowledge differed significantly between groups (F(2,143) = 3.31, p = 0.039), with pediatricians outscoring family medicine physicians by 9.39% (p = 0.035); vignette knowledge, mandatory reporting, the combined multivariate ANOVA (MANOVA), and both confidence attitude questions (AQ4, AQ5) showed no significant specialty differences.

Domain	ANOVA F (df)	p-value	Significant Pairwise Differences (Tukey)
Overall Knowledge	3.31 (2,143)	0.039	Pediatrics > Family Medicine by 9.39% (p = 0.035)
Vignette Knowledge	1.89 (2,143)	0.155	None
Mandatory Reporting	2.59 (2,143)	0.078	None
Combined (MANOVA)	Wilks’ λ = 0.9465	0.098	None (Roy’s largest root marginal p = 0.034)
AQ4 - Confidence in Diagnosis	1.81 (2,142)	0.17	None
AQ5 - Confidence in Reporting	0.18 (2,142)	0.83	None

Vignette-based knowledge questions

For vignette-based knowledge questions that assessed physicians’ ability to differentiate between measles, rubella, and roseola, pediatrics had the highest score. However, the ANOVA results and Tukey post hoc comparisons revealed no significant difference between specialties when vignette-based knowledge questions were examined separately (F(2,143) = 1.89, p = 0.15) (Table 3, Table 4).

**Table 4:** Vignette and Mandatory Reporting Specific Knowledge Scores by Specialty Comparison of clinical vignette-based and mandatory reporting knowledge scores among physician respondents, stratified by medical specialty (146), presented as mean percentage scores and standard deviations. ¹ Vignette-based knowledge questions assessing the ability to differentiate measles, rubella, and roseola. ² Mandatory reporting knowledge questions assessing understanding of reporting requirements.

Specialty	n	Vignette Knowledge Mean (%)	SD (%)	Mandatory Reporting Mean (%)	SD (%)
Family Medicine	48	65.0¹	27.1¹	78.3²	23.0²
Internal Medicine	24	72.6¹	28.0¹	79.7²	25.1²
Pediatrics	74	75.2¹	27.2¹	89.3²	16.4²
Total	146	71.4	27.5	84.1	20.9

Mandatory reporting knowledge questions

Similarly, when knowledge questions about mandatory reporting requirements were examined separately, results showed no significant differences among the three specialties. Although overall ANOVA approached significance (F(2,143) = 2.59, p = 0.08), the Tukey pairwise comparisons were not significant (Table 3, Table 4).

To evaluate whether specialty had a combined effect on the diagnosis of VE and mandatory reporting knowledge, a multivariate analysis of variance (MANOVA) was conducted. Results demonstrated no statistically significant difference as indicated by Wilks’ lambda (lambda = 0.9465, F = 1.98, p = 0.098), Pillai's trace (p = 0.10), Lawley-Hotelling trace (p = 0.10), and Roy's largest root (p = 0.034) (Table 3). While Roy’s largest root was marginally significant, this result can not be supported by the other indices, which showed no significant difference.

Global physician attitudes

Physician opinions on the complexity and time burden associated with diagnosing VE (attitude question, AQ2) were relatively evenly distributed, with 91 (56.17%) of respondents indicating that the diagnostic workup of VE was complex and time-consuming, whereas 71 (43.83%) indicated otherwise (Figure 2). Regarding interest in increased training on the workup and management of VE (AQ3), a substantial majority, 148 (91.36%), expressed a desire for additional training. This finding is consistent with perceptions of diagnostic inadequacy of VE (AQ1), where 43 (26.54%) of respondents felt that VE were certainly underdiagnosed or misdiagnosed, while 1 (0.62%) believed they were not.

**Figure 2:**
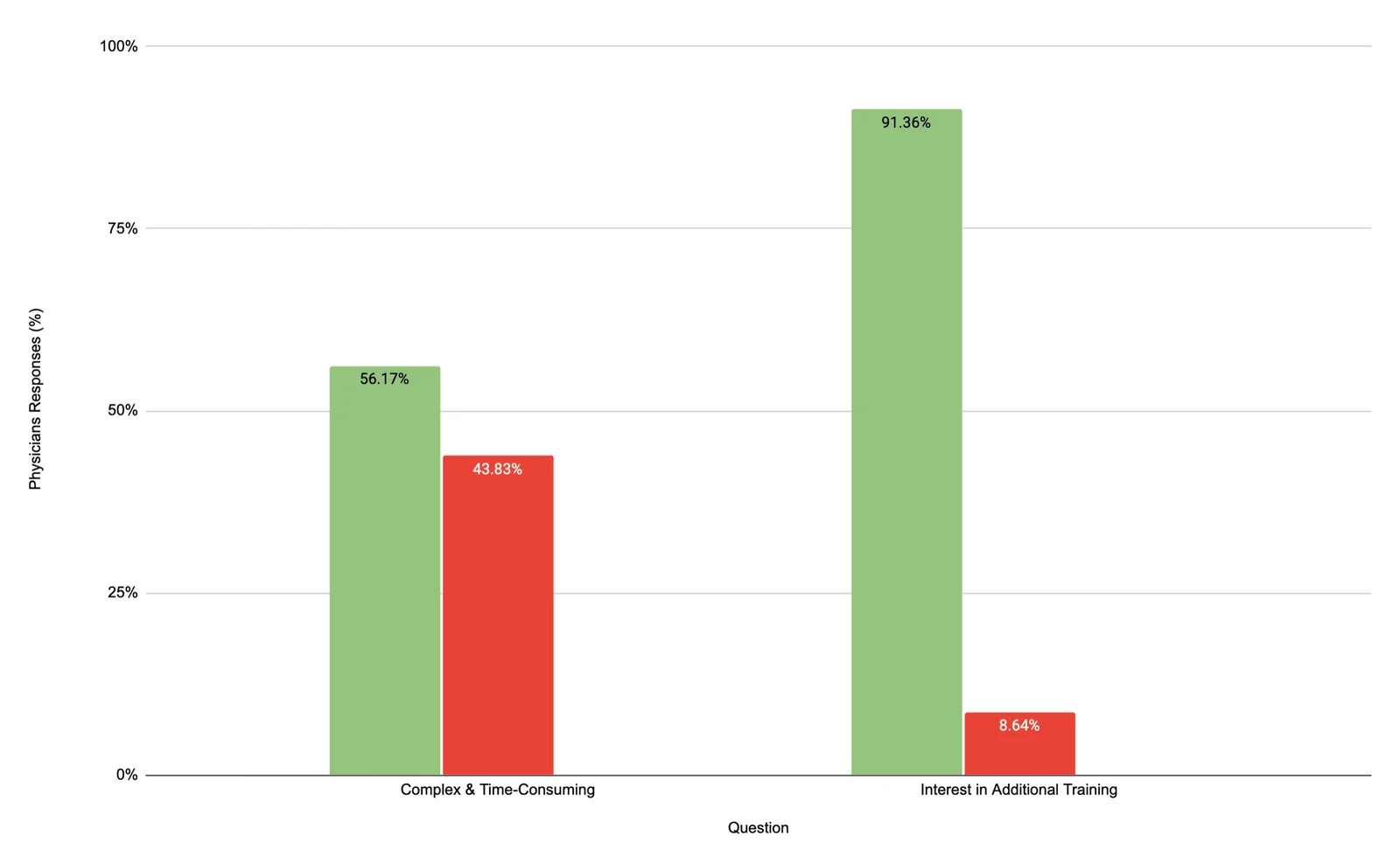
Global Physician Attitudes Responses Distribution of responses to the global attitude questions (yes/no) on the complexity and time burden of diagnosing viral exanthem (VE), interest in additional training, and perceived under- or misdiagnosis; green bars indicate "yes" responses and red bars "no."

Specialty-based differences in attitudes

Physicians’ confidence in diagnosing VE (AQ4) and properly reporting a diagnosis of rubella, varicella, measles, and roseola to public health authorities was evaluated for specialty-based differences in attitudes (AQ5). The ANOVA for AQ4 yielded F(2,142) = 1.81, p = 0.17 and F(2,142) = 0.18, p = 0.83 for AQ5 (Table 3). These results showed that neither attitude question had a significant difference between specialties. Additionally, post hoc comparisons were not significant. 

Associations between knowledge scores and physician attitudes

Two linear regression models were used to evaluate the relationship between knowledge scores and physician attitudes. The first linear regression model evaluated whether physicians’ confidence in diagnosing VE predicted vignette-based knowledge questions (AQ4). Results showed a significant positive relationship: with every one-point increase in positive attitude, vignette-based knowledge scores increased by 10.73% (F(1,143) = 12.93, p < 0.001) (Table 5).

**Table 5:** Linear Regression of Physician Attitudes Predicting Knowledge Scores Two linear regression models tested whether physician confidence predicted knowledge. Greater confidence in diagnosing viral exanthem (VE) (AQ4) predicted higher vignette-based knowledge scores (β = 10.73%, F(1,143) = 12.93, p < 0.001), and greater confidence in reporting requirements (AQ5) predicted higher mandatory reporting knowledge scores (β = 9.68%, F(1,143) = 20.40, p < 0.001).

Outcome	Predictor	β (%)	F (df)	p-value
Vignette-based knowledge score	Confidence in diagnosing viral exanthems (AQ4)	10.73	12.93 (1,143)	<0.001
Mandatory reporting knowledge score	Confidence in mandatory reporting requirements (AQ5)	9.68	20.40 (1,143)	<0.001

The second linear regression model examined the relationship between physicians’ perceived confidence in mandatory reporting requirements and mandatory reporting knowledge scores. Results also showed a significant positive relationship: with every one-point increase in positive attitude, mandatory reporting knowledge scores increased 9.68% (F(1,143) = 20.40, p < 0.001) (Table 6).

**Table 6:** 95% Confidence Intervals for Mean Overall Knowledge Scores by Specialty Group Overall knowledge scores with 95% Wilson confidence intervals and asymmetric error margins by specialty: family medicine (73.26%, n = 48; 59.37-83.71%), internal medicine (76.39%, n = 24; 56.56-88.94%), pediatrics (82.66%, n = 74; 72.48-89.61%), and the total sample (78.54%, n = 146; 71.19-84.42%).

Group	n	Mean (%)	SD (%)	SE	Lower 95% CI	Upper 95% CI
Family Medicine	48	73.26	22.74	3.28	66.66	79.86
Internal Medicine	24	76.39	25.02	5.11	65.82	86.96
Pediatrics	74	82.66	16.42	1.91	78.86	86.46
Overall	146	78.54	20.53	1.7	75.18	81.9

The responses from physicians who diagnosed measles, rubella, and roseola during their careers were evaluated. It was found that roseola was the most frequently diagnosed VE, with a 107 (66%) response rate. This was followed by measles, reported by 49 (30%) respondents, and rubella, reported by 28 (17%) respondents (Figure 3).

**Figure 3:**
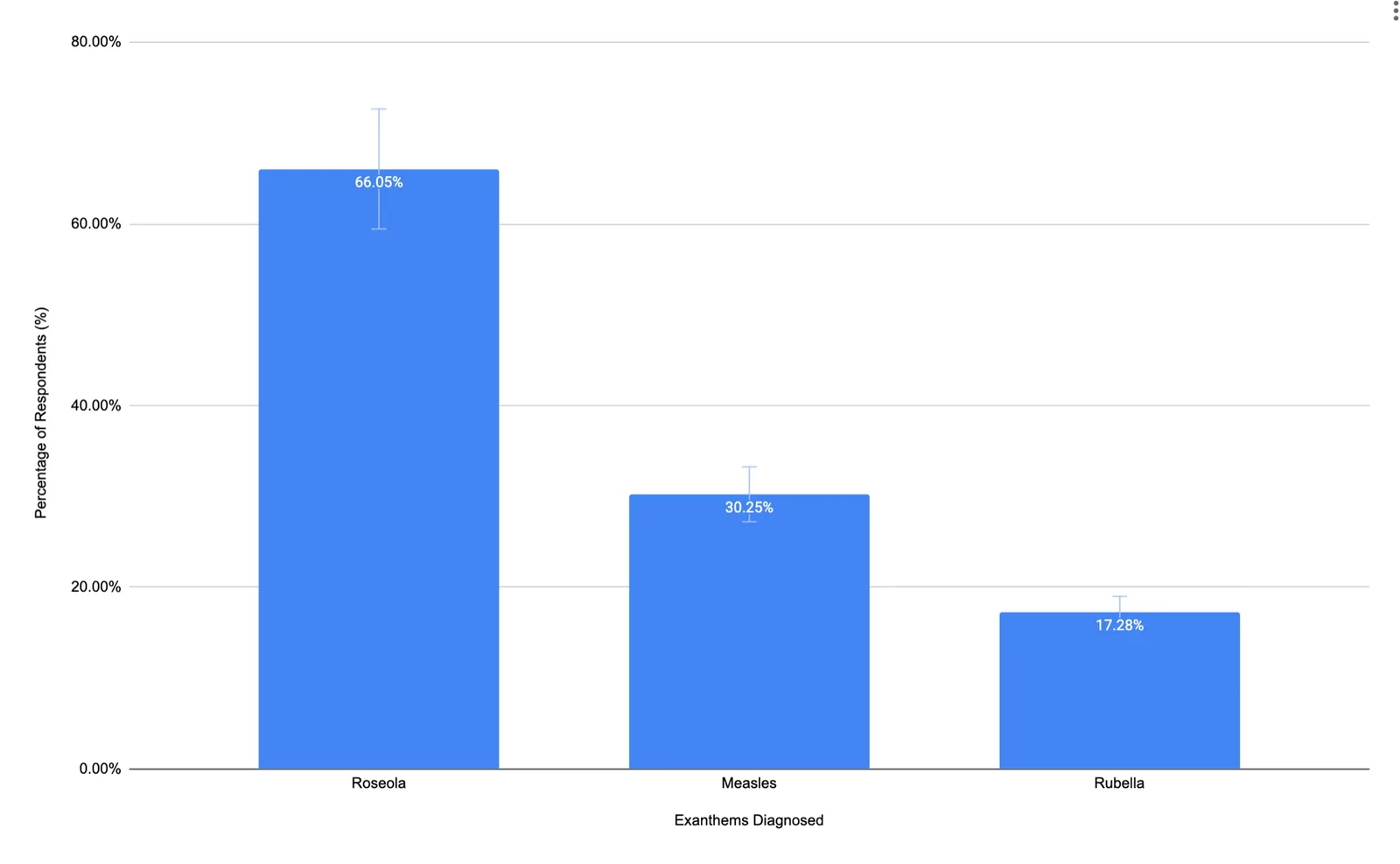
Self-Reported Clinical Practice Diagnosis of Viral Exanthems Among Physician Respondents Bars represent the percentage of respondents who reported having ever diagnosed each viral exanthem during their clinical career.

Furthermore, perceived vaccination rates were evaluated based on physicians' responses. Eighty-five (52.47%) physicians stated that less than 10% of children were vaccinated in their area of practice, 30 (18.52%) stated that 10-20% of children were vaccinated, five (3.09%) stated that 21-30% of children were vaccinated, and seven (4.32%) stated that more than 30% of children were vaccinated (Figure 4).

**Figure 4:**
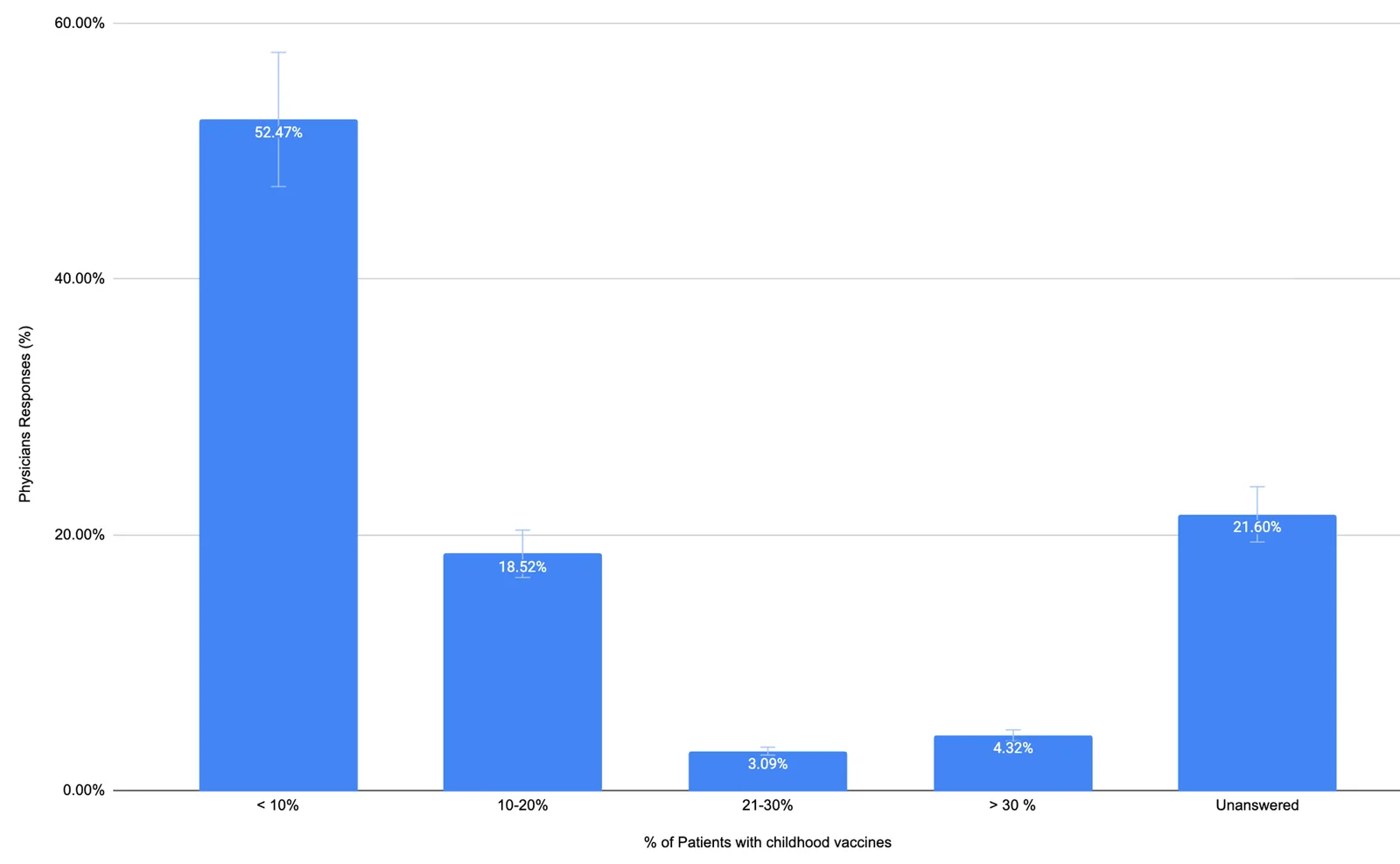
Perceived Childhood Vaccination Rates Comparison of physician-reported perceptions of childhood vaccination rates in their practice areas across family medicine, internal medicine, and pediatrics.

## Discussion

In this survey, physicians practicing in Florida were assessed for their knowledge, attitudes, and opinions related to the diagnostic workup of VE and clinical practices pertaining to VE (measles, rubella, roseola). Physician responses across these domains revealed knowledge gaps and limited confidence in the diagnosis and mandatory reporting of VE. Our study confirms that respondents reported diagnosing measles, rubella, and roseola during their careers, highlighting the continued and emerging public health relevance of viral exanthems in the southeastern United States.

The knowledge-based question on which physicians demonstrated the lowest performance involved diagnosing rubella from a clinical vignette, whereas the highest-scoring question addressed mandatory reporting guidelines of rubella. These findings suggest that physicians may be familiar with reporting once viral exanthem diagnoses are made, but clinical differentiation of similar rash presentations remains a challenge. Among specialties, pediatricians achieved the highest mean score, followed by internal medicine (IM) and family medicine (FM). This distribution is consistent with the epidemiology of VE, which is commonly present in the pediatric population [20]. Mean knowledge scores were statistically comparable among specialties, with the exception of pediatricians, who had a statistically significantly higher mean score than FM physicians. This finding was somewhat unexpected, as ambulatory pediatricians and FM physicians are typically the first to encounter VE cases, while involvement of an IM or infectious disease (ID) physician more often requires a referral or for the patient to first be seen by an emergency medicine physician.

Although specialty-based differences in vignette-based and mandatory reporting knowledge were not statistically significant when examined independently, there was an overall trend toward lower performance among non-pediatric specialties. This suggests that experience and clinical exposure may influence applied knowledge. This could be attributed to multiple reasons. One reason is that pediatricians and infectious disease physicians have more exposure to VE and thus must be constantly up-to-date on regulations and mandatory reporting. Importantly, multivariate analyses did not demonstrate a combined specialty effect on diagnostic and reporting knowledge, indicating that gaps are present across disciplines rather than limited to a single specialty.

Findings on physician attitudes further contextualize these findings. The majority of respondents reported feeling “somewhat confident” in diagnosing and reporting VE. Self-reported confidence was positively associated with knowledge scores, suggesting some concordance between perceived and measured knowledge. These findings, in conjunction with the near-unanimous physician desire for increased training in addressing and managing VE in Florida, underscore perceived knowledge deficits and discrepancies. These findings support evaluating targeted educational interventions in future studies. Additionally, incorporating refresher modules or didactic content within continuing medical education may meaningfully improve outcomes and mandatory reporting practices.

The trend of declining vaccination rates has been an ongoing topic of concern for years. Vaccination coverage has been experiencing a trending decline in Florida among kindergartners since the 2018-2019 school year, with coverage for school-mandated vaccines dropping below 90% in the 2024-2025 school year, undermining herd immunity [21]. Our study shows that, correspondingly, some vaccine-preventable diseases have seen an increase in the United States in recent years. The most memorable instance has been the recent surge of measles cases across the country, predominantly in areas with lower vaccination rates [16]. Also notable has been the increase in pertussis in 2024 and 2025, with reported cases in 2024 being six times higher than pre-pandemic 2019 rates [22]. Other vaccine-preventable diseases, including mumps, toxigenic C. diphtheria, rubella, invasive pneumococcal disease, and *Haemophilus influenzae *type B-associated epiglottitis, have not reported significantly increased national rates, which may be attributed to high overall vaccination rates [23, 24, 25, 26, 27]. These trends are a warning to practicing health professionals across the country and possibly threaten major setbacks for public health. Due to vaccine mandates, newer generations of Florida physicians who began practicing in this century have encountered the lowest number of cases of vaccine-preventable disease in United States history, leading to concerns regarding lack of experience in recognition and management of these diseases [28, 29].

Limitations

This study has potential limitations that should be considered. These limitations are primarily methodological. First, the process of participant recruitment relied on email addresses provided publicly by the Florida Department of Health practitioner database, with a direct focus on FM, pediatrics, and infectious disease physicians. IM physicians were not initially targeted during the email collection process. Instead, they were identified through subspecialty categorization of existing survey responses. This represents a sampling limitation that may have contributed to uneven specialty representation and response rates.

Secondly, the study focused mainly on FM, pediatrics, and IM, which may not be fully representative of the knowledge and practices of VE across all specialties, such as emergency medicine, dermatology, and obstetrics, where VE may also be encountered. Therefore, the study’s findings are more reflective of primary-care-based settings. Responses were also obtained from multiple metropolitan regions across Florida. This may result in the overall response rate being limited, and some counties were minimally represented. This geographic imbalance may limit the generalizability of findings to all practice settings, particularly in rural or underserved areas. The low overall response rate relative to the contacted pool of physicians raises the possibility of nonresponse bias, potentially overrepresenting higher knowledge levels and more favorable attitudes. Additionally, the unequal distribution of respondents across specialty groups affects the statistical power of pairwise comparisons and may reduce the ability to detect true differences between smaller groups.

Additional limitations include the cross-sectional design of the study, which allows for identification of associations but does not allow causality to be established. Since the survey participation was voluntary through publicly available physician contact information, this may have introduced selection and nonresponse bias. Several measures of the study, such as physician confidence, prior diagnoses, and self-perceived vaccination rates, were self-reported; therefore, this may be subject to recall or reporting bias. 

Future directions

Future research should explore physician-reported barriers to VE diagnosis and mandatory reporting guidelines to identify areas for intervention, which may include structured continuing medical education modules on the clinical differentiation of exanthems with similar clinical presentations. The same applies to a broader, national version of this study with a larger sample size and a more robust dataset. A Florida county-based analysis using mis/underdiagnosis rates of VE could aid in identifying high-risk areas in particular need of targeted public health and healthcare outreach to improve awareness, early recognition, and management of VE. A similar analysis could be conducted by analyzing the correlation between VE knowledge in rural and urban practice settings. Additionally, it would be interesting to see whether there is any association between years of practice and accuracy of VE diagnosis, particularly for physicians in practice prior to the adoption of mandatory school vaccines by Florida.

## Conclusions

The study evaluates physician knowledge, practice, and attitudes regarding viral exanthem diagnosis and mandatory reporting requirements in Florida. Respondents demonstrated moderate-to-high overall knowledge, with lower performance on clinical differentiation than on mandatory reporting. There was a strong statistical correlation between knowledge scores and physician confidence in diagnosis, underlining the importance of additional training for more accurate diagnosis and comprehensive management.

Additionally, knowledge gaps were identified, particularly in the clinical differentiation of rubella. These knowledge gaps are present in all specialties, demonstrating a systemic nature rather than confinement to a single discipline.

This topic is currently clinically relevant, as declining vaccination rates and increasing measles outbreaks carry important public health implications. Therefore, early diagnosis and reporting of VE are critical for outbreak prevention and control. Future studies are necessary on a larger scale, in different geographical areas, and among other physician specialties to evaluate differences, identify shared patterns, and address knowledge gaps in viral exanthem diagnosis.

## Appendices

**Table 7:** Viral Exanthem Knowledge, Attitudes, and Practices (KAP) Questionnaire. This 19-question REDCap survey instrument was developed by the study authors to assess physician knowledge, attitudes, and practices regarding viral exanthem diagnosis and mandatory reporting requirements. Credits: Winnie Chen, Sarah Ballarin, Maria Fioletova, Chanakya R. Bhosale, Thomas Matthews, Cyril Blavo, Jayson Forbes, and Anna Potter. The instrument is the authors’ original creation.

Domain	#	Question	Answer Choices	Correct Answer
Knowledge	1	A 32-year-old female presents with a mild fever and rash on the face for 1 day followed by the trunk and extremities, along with a mild sore throat for 3 days. She also has apparent posterior cervical and posterior auricular lymphadenopathy. What is the most likely diagnosis?	A) Measles B) Scarlet Fever C) Rubella D) Chickenpox	C
	2	As a physician, which of the following statements about rubella reporting guidelines do you consider true?	A) Rubella is a reportable disease in all states and cases should be reported to local health departments within 24 hours of diagnosis B) Physicians are not required to report rubella cases if the patient has been vaccinated C) Only laboratory-confirmed cases of rubella need to be reported, while clinical cases can be excluded D) Reporting rubella cases is only necessary during outbreaks, and individual cases do not need to be reported	A
	3	A 10-year-old male is brought in for a rash that began today after a prodrome of fever, itchy eyes, and rhinorrhea. He recently took amoxicillin for a sore throat, and vaccination records are unavailable. He is febrile but vital signs are otherwise stable. Examination reveals a maculopapular rash on the face. Oropharyngeal findings are depicted in the photo. Which diagnosis is most likely?	A) Varicella virus B) Measles virus C) Parvovirus B19 D) Adverse effect of medication E) Epstein-Barr virus	B
	4	After a diagnosis of measles is made, which of the following is the best next step in management?	A) Optional reporting to public health authority (e.g., Department of Health) B) Mandatory reporting to public health authority the same business day C) Mandatory reporting to public health authority by the next business day D) Mandatory reporting to public health authority within 1 week	B
	5	A 2-year-old male who attends daycare comes to the clinic with a nonpruritic rash that began on the trunk. Over the past couple of days, the patient has had rhinorrhea and has been crying more frequently. He developed a high fever for three days that broke yesterday. Examination shows mild intercostal retraction but lungs are clear to auscultation. Inspection reveals an erythematous maculopapular rash diffusely distributed across the trunk and the entirety of all extremities. Which of the following is the most likely cause?	A) Parvovirus B19 B) Roseola C) Rubella D) Measles E) Scarlet fever F) Pityriasis rosea	B
	6	After a diagnosis of roseola is made, which of the following is the best next step in management?	A) Optional reporting to public health authority (e.g., Department of Health) B) Mandatory reporting to public health authority the same business day C) Mandatory reporting to public health authority by the next business day D) Mandatory reporting to public health authority within 1 week	A
Attitude	7	In your opinion, to what extent are roseola, rubella, and/or measles misdiagnosed and/or underdiagnosed in Florida?	A) Not at all B) Probably misdiagnosed C) Probably underdiagnosed D) Certainly underdiagnosed E) Certainly misdiagnosed	—
	8	In your opinion, is the process required to confirm rubella, varicella (chickenpox), measles, or roseola in your practice setting complex and time-consuming?	A) Yes B) No	—
	9	In your opinion, do you think there is a need for more training on screening, diagnosis, treatment, and management of rubella, varicella (chickenpox), measles, or roseola in your area or state?	A) Yes B) No	—
	10	Which of the following have you diagnosed? (check all that apply)	A) Measles B) Rubella C) Roseola/HHV-6 D) N/A	—
	11	Approximately what percentage of your patients do not receive routine childhood vaccinations (e.g., measles, mumps, rubella, DTaP, IPV, hepatitis B)?	A) <10% B) 10–20% C) 21–30% D) Over 30% E) I don't know	—
	12	How confident do you feel diagnosing rubella, varicella (chickenpox), measles, and roseola?	A) Not at all B) Somewhat confident C) Confident D) Very confident	—
	13	How confident do you feel about properly reporting to public health authorities a diagnosis of rubella, varicella (chickenpox), measles, or roseola when the need arises?	A) Not at all B) Somewhat confident C) Confident D) Very confident	—
Demographics	14	Do you practice in North Florida or South Florida?	A) North Florida B) South Florida	—
	15	How many years have you been in practice (i.e., how many years since you finished residency)?	Free-text numeric response	—
	16	Which health profession category best describes you?	A) Osteopathic physician (DO) B) Allopathic physician (MD) C) DO resident D) MD resident	—
	17	In which of the following residencies and/or fellowships did you train? (check all that apply)	A) Pediatrics residency B) Internal medicine residency C) Family medicine residency D) Infectious disease residency E) Cardiology fellowship F) Hematology/oncology fellowship G) Gastroenterology fellowship H) Nephrology fellowship I) Rheumatology fellowship	—
	18	What is the geographic setting of your practice? (check all that apply)	A) Rural B) Urban C) Sub-urban	—
	19	What is the business setting of your practice? (check all that apply)	A) Academic B) Private practice C) Community hospital/clinic D) Telemedicine E) Other	—
